# Supplementary material for: On-Chip Microwave Sensing of Nonequilibrium Quasiparticles in α‑Tantalum Superconducting Circuits on Silicon for Scalable Quantum Technologies
Source: ACS Appl Mater Interfaces. 2026 Jan 5;18(1):3007–13. doi: 10.1021/acsami.5c18323 (PMC12781050; doi:10.1021/acsami.5c18323)
Supplement: Supplementary file 1 [file am5c18323_si_001.pdf]

Supplementary Information

On-chip microwave sensing of nonequilibrium quasiparticles in α-tantalum superconducting circuits on silicon for scalable quantum technologies

Shima Poorgholam-Khanjari, Paniz Foshat,
Mingqi Zhang, Valentino Seferai, Martin Weides, and Kaveh Delfanazari*

Electronics and Nanoscale Engineering Division, James Watt School of Engineering, University of Glasgow, Glasgow, UK

*Corresponding author: kaveh.delfanazari@glasgow.ac.uk

S1 Calculation of $< n_{ph} >$

The photon number inside the $\frac{\lambda}{4}$ resonator can be determined by [1]:

$$P_{\text{in}} = P_{\text{trans}} + P_{\text{ref}} + P_{\text{abs}}$$

$$P_{\text{ref}} = P_{\text{in}}(|S_{11}|^2)$$

$$P_{\text{trans}} = P_{\text{in}}(|S_{21}|^2)$$

$$P_{\text{abs}} = P_{\text{in}}(1 - |S_{21}|^2 - |S_{11}|^2)$$

$$< n_{ph} >= \frac{2Q_c}{\omega_0}\left(\frac{Q_i}{Q_i + Q_c}\right)^2 \frac{P_{\text{in}}}{\hbar\omega_0} \tag{S1}$$

Where P_{in} is the input power at the resonator, calculated as

$$P_{\text{in}} = P_{\text{VNA}} + P_{\text{fridge att}} + P_{\text{RT att}},$$

in which $P_{\text{fridge att}}$ is the attenuation inside the fridge, and $P_{\text{RT att}}$ is the room temperature attenuation. P_{ref} is the reflected power, P_{trans} is the transmitted power, and P_{abs} is the power absorbed by the resonator.

S2 Extraction of TLS loss parameters

The common TLS model is defined by [2]:

$$\delta_{TLS}(T, P) = \frac{1}{Q_{\text{TLS}}} = \frac{1}{Q^0_{\text{TLS}}} \frac{\tanh(\frac{hf_r}{2K_BT})}{\sqrt{1 + \left(\frac{<n_{\text{ph}}>}{n_{\text{c}}}\right)^\beta}} \tag{S2}$$

The TLS loss at zero power and temperature ($< n_{ph} >$ = 0 and T= 0) is given by $\frac{1}{Q^0_{TLS}}$= δ^0_{TLS}. Here, n_c represents the critical photon number, $< n_{ph} >$ is the average photon number, and β is known to be design-dependent [3, 4]. We fitted the measured data using Eq. (S2) for all resonance frequencies at the base temperature $T = 77$ mK. For f_r = 3.654 GHz, we obtained $\frac{1}{Q^0_{TLS}}$ = 6.11 $\times 10^{-6}$ and β = 0.44, which indicates the strength of TLS saturation with power reflecting how effectively the applied power can suppress TLS-induced losses.

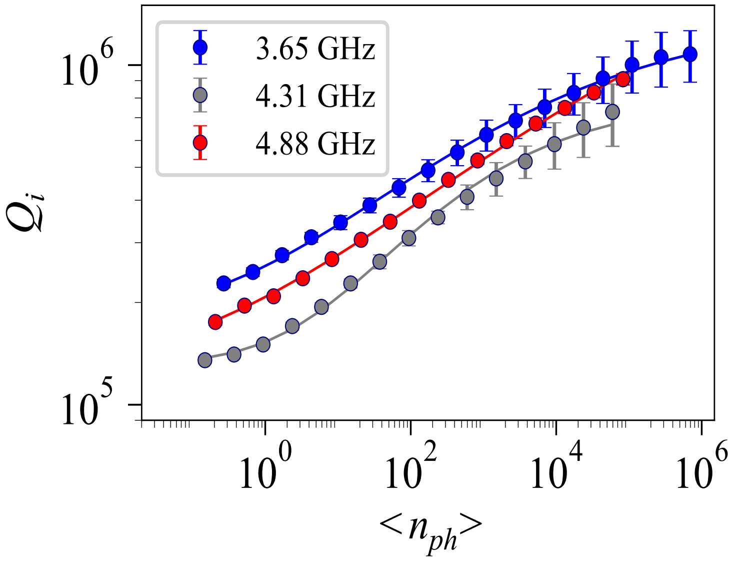

Figure S1: Internal quality factor (Q_i) of three CPW resonators on silicon as a function of average photon numbers $< n_{ph} >$, the scatter plots are for measurement data, solid lines are fitted data based on Eq. S2 and error bars are depicted with caps.

S3 Microwave spectroscopy setup

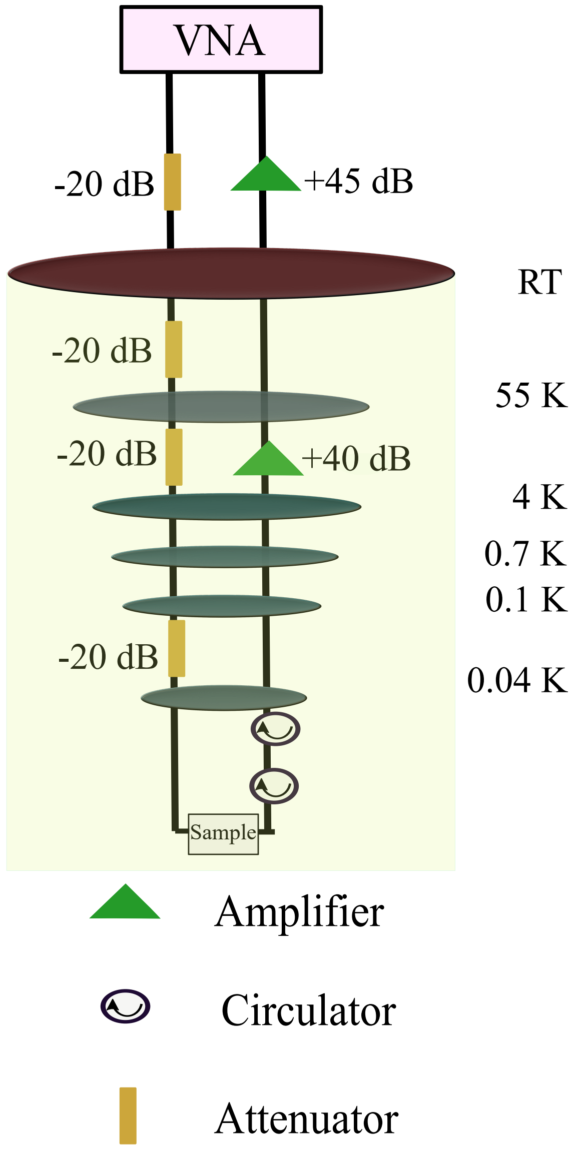

Figure S2: Schematic of the cryogenic setup for sub-Kelvin microwave spectroscopy of the chip.

References

[1] C. X. Yu, S. Zihlmann, G. Troncoso Fernández-Bada, J.-L. Thomassin, F. Gustavo, É. Dumur, and R. Maurand, "Magnetic field resilient high kinetic inductance superconducting niobium nitride coplanar waveguide resonators," *Applied Physics Letters*, vol. 118, no. 5, 2021.

[2] J. Van Damme, T. Ivanov, P. Favia, T. Conard, J. Verjauw, R. Acharya, D. Perez Lozano, B. Raes, J. Van de Vondel, A. Vadiraj, *et al.*, "Argon-milling-induced decoherence mechanisms in superconducting quantum circuits," *Physical Review Applied*, vol. 20, no. 1, p. 014034, 2023.

[3] J. Goetz, F. Deppe, M. Haeberlein, F. Wulschner, C. W. Zollitsch, S. Meier, M. Fischer, P. Eder, E. Xie, K. G. Fedorov, *et al.*, "Loss mechanisms in superconducting thin film microwave resonators," *Journal of Applied Physics*, vol. 119, no. 1, 2016.

[4] F. W. Carter, T. Khaire, C. Chang, and V. Novosad, "Low-loss single-photon nbn microwave resonators on si," *Applied Physics Letters*, vol. 115, no. 9, 2019.
